# Sequence and organisation of the mitochondrial genome of Japanese Grosbeak (*Eophona personata*), and the phylogenetic relationships of Fringillidae

**DOI:** 10.3897/zookeys.995.34432

**Published:** 2020-11-18

**Authors:** Guolei Sun, Chao Zhao, Tian Xia, Qinguo Wei, Xiufeng Yang, Shi Feng, Weilai Sha, Honghai Zhang

**Affiliations:** 1 College of Life Science, Qufu Normal University, Qufu, Shandong province, China Qufu Normal University Qufu China

**Keywords:** *Eophona personata*, gene order, mitochondrial genome, phylogenetic analysis

## Abstract

Mitochondrial DNA is a useful molecular marker for phylogenetic and evolutionary analysis. In the current study, we determined the complete mitochondrial genome of *Eophona personata*, the Japanese Grosbeak, and the phylogenetic relationships of *E. personata* and 16 other species of the family Fringillidae based on the sequences of 12 mitochondrial protein-coding genes. The mitochondrial genome of *E. personata* consists of 16,771 base pairs, and contains 13 protein-coding genes, 22 transfer RNA (tRNA) genes, 2 ribosomal RNA (rRNA) genes, and one control region. Analysis of the base composition revealed an A+T bias, a positive AT skew and a negative GC skew. The mitochondrial gene order and arrangement in *E. personata* was similar to the typical avian mitochondrial gene arrangement. Phylogenetic analysis of 17 species of Fringillidae, based on Bayesian inference and Maximum Likelihood (ML) estimation, showed that the genera *Coccothraustes* and *Hesperiphona* are closely related to the genus *Eophona*, and further showed a sister-group relationship of *E. personata* and *E. migratoria*.

## Introduction

*Eophona personata* (Passeriformes: Fringillidae), commonly known as the Japanese Grosbeak, is a granivorous passerine with the adults reaching a size of ca. 23 cm. The species is mainly distributed in Far Eastern Asia including Eastern Siberia, Northeast China, North Korea, and Japan. Grosbeaks are migratory birds and move to South China during winter (Clement et al. 1993). Grosbeaks primarily feed on seeds, with preference for certain plants, and insects (Kominami 1987); for example, the seeds of *Celtis* and *Aphananthe* are favored during autumn and winter (Nimura 1993; Yoshikawa and Kikuzawa 2009).

The mitochondrial genome (hereafter mitogenome) is a useful molecular marker for phylogenetic analysis, and is widely used in the evolutionary analysis of a variety of species (Anderson et al. 1981; Boore 1999; Sun et al. 2016). The animal mitogenome is usually a short, closed, circular, double stranded molecule, and comprises of 37 genes: 13 protein-coding genes (PCGs; *ND1*, *ND2*, *ND3*, *ND4*, *ND4L*, *ND5*, *ND6*, *COI*, *COII*, *COIII*, *ATP6*, *ATP8*, *CYTB*), two rRNA genes (12S rRNA and 16S rRNA), and 22 tRNA genes. Compared to nuclear DNA, the mitogenome is a more conserved molecule, and is maternally transmitted (Wolstenholme 1992; Boore 1999; da Fonseca et al. 2008).

With ca. 6000 species, passerines account for more than half of the total number of extant birds (Gill et al. 2020). Based on the analysis of hundreds of bird mitochondrial data, at least seven gene arrangements have been found (Zhou et al. 2014). Gene rearrangements in passerines are very common, and most reported passerines have the standard gene order consistent with chickens *Gallus gallus* (Desjardins and Morais 1990). In addition, Passeriformes have at least four gene arrangement (Harrison et al. 2004). The different rearrangements involve the initial tandem duplication, and the partial loss of the segment containing the control region (CR) (Caparroz et al. 2018).

The passerine family Fringillidae comprises ca. 50 genera and 230 species (Gill et al. 2020). The phylogenetic relationships between the species in the family Fringillidae and the superfamily Passeroidea are incompletely resolved (Van der Meij et al. 2005). Previous phylogenetic studies of Fringillidae have clarified several aspects of their relationships (Arnaiz-Villena et al. 2001; Van der Meij et al. 2005; Lerner et al. 2011; Zuccon et al. 2012; Tietze et al. 2013; Sangster et al. 2015; Zhao et al. 2016). The aim of this study is to determine the complete mitogenome of *E. personata* and provide a mitogenomic perspective on the phylogenetic relationships of *E. personata* and 16 other species of the family Fringillidae.

## Materials and methods

### Sample collection and DNA extraction

The specimen of *E. personata*, which had died due to poaching activities, was collected from Shenyang City, Liaoning Province, China, and was stored in the laboratory and then frozen to -80 °C before further processing and analysis. All experiments involving animals were approved by the Qufu Normal University Institutional Animal Care and Use Committee (Permit number: QFNU2018-010) and executed in accordance with the Guide to Animal Experiments of the Ministry of Science and Technology (Beijing, China). DNA was extracted from muscle tissue using the DNeasy Blood and Tissue Kit (Qiagen, Hilden, Germany) following the manufacturer’s protocol.

### Cloning and sequencing of mitochondrial genome

Primers were designed based on mitogenome sequences of a sparrowhawk (*Accipiter nisus*, GenBank accession number KM360148) and Chinese Grosbeak *E. migratoria* (KX423959). MEGA5.1, Primer Premier 6.0, and the NCBI-Primer blast database (http://www.ncbi.nlm.nih.gov/BLAST) were used to determine the primers. All the amplified fragments were separated by gel electrophoresis and purified by Agarose Gel Extraction Kit (Qiagen). Purified fragments were cloned into PMD18-T vectors, and transformed into competent *E. coli* cells. Positive clones were identified by blue-white screening and sequenced. Bidirectional sequencing was conducted at Sangon Biotech (Shanghai) using the ABI 3730xl DNA Analyzer (PE Applied Biosystems, San Francisco, CA, USA).

### Bioinformatics analysis and statistical procedures

The raw sequences were assembled using the software programs BioEdit (version 7.2. 5) and Chromas Pro 1.7.7 (Goodstadt and Ponting 2001). Genes were identified by aligning the identified sequences with the known sequence of the mitogenome of *E. migratoria*. The tRNA gene structure was predicted using Scan-SE 1.21 (http://lowelab.ucsc.edu/tRNAscan-SE) and ARWEN (http://130.235.46.10/ARWEN/) (Lowe and Eddy 1997).

Phylogenetic analysis of 17 Fringillidae species (accession numbers are provided in Table 2) and *Phasianus colchicus* (NC_015526) was conducted using Bayesian inference and Maximum Likelihood (ML) estimation based on the sequences of 12 mitochondrial PCGs; *ND6* gene, which is encoded on the L-strand, was excluded from analysis. The sequences were aligned with ClustalX 1.81 (Thompson et al. 1997; Jeanmougin et al. 1998), and phylogenetic analysis was performed using MrBayes 3.1.2 and PAUP* 4.0 (Swofford 2001; Ronquist and Huelsenbeck 2003).

Based on the Akaike Information criterion (AIC), GTR+I+G was estimated as the best-fit substitution model using Modeltest 3.7 (Posada and Crandall 1998). Bootstrap tests were based on 1000 replicates (Felsenstein 1981; Wilgenbusch and Swofford 2003). For Bayesian analysis, Metropolis-coupled Markov chain Monte Carlo analysis was performed, with 4 chains run in parallel, for 2,000,000 generations, and the first 25% of each of the sampled 1000 generations were excluded as burn-in.

Following Sangster and Luksenburg (2020), we verified the identity and integrity of our mitogenome sequence of *E. personata* with reference sequences of multiple protein-coding genes: NADH dehydrogenase subunit 2 (ND2, 1041 bp; n = 1), part of cytochrome oxidase subunit I (COX1, 698 bp; n = 6), and cytochrome *b* (CYTB, 1143 bp; n = 2). These markers are commonly used in avian systematics, and reference sequences were available for each marker.

## Results

### Comparison of mitogenomes of Japanese grosbeak and those of other species of Fringillidae

**Figure 1.**
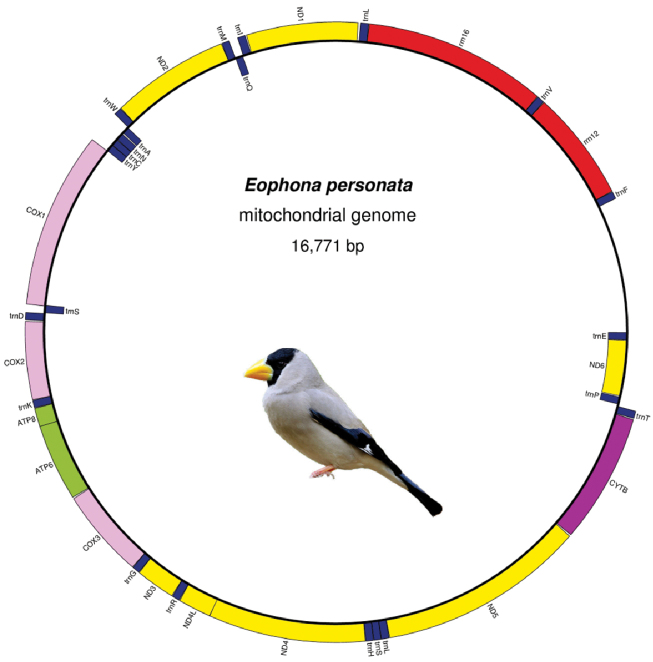
Circular map of the mitochondrial genome of *Eophona personata*. tRNAs are denoted as one-letter symbols according to IUPAC-IUB single-letter amino acid codes; L1 = UUR, L2 = CUN, S1 = UCN, S2 = AGY.

The complete mitochondrial genome of *E. personata* (KX812499, GenBank) was sequenced and a genome map was constructed (Fig. 1). The genome contains 13 PCGs, 22 transfer RNA (tRNA) genes, two ribosomal RNA (rRNA) genes, and one control region (Table 1).

**Table 1.** Organization of the mitochondrial genome of *Eophona personata*.

Gene	Location (bp)	Size (bp)	Spacer (+) or overlap (–)	Strand	Start codon	Stop codon	Anticodon
**D-loop**	1–1187	1187		H	–	–	–
**tRNA^Phe^**	1188–1255	68	0	H	–	–	GAA
**12S rRNA**	1256–2230	975	0	H	–	–	–
**TRNA^Val^**	2231–2300	70	0	H	–	–	TAC
**16S rRNA**	2301–3904	1604	0	H	–	–	–
**tRNA^Leu(UUR)^**	3905–3979	75	16	H	–	–	TAA
**ND1**	3996–4973	978	6	H	ATG	AGG	–
**tRNA^Ile^**	4980–5053	74	3	H	–	–	GAT
**tRNA^Gln^**	5057–5127	71	-1	L	–	–	TTG
**tRNA^Met^**	5127–5195	69	0	H	–	–	CAT
**ND2**	5196–6236	1041	-1	H	ATG	TA-	–
**tRNA^Trp^**	6236–6305	70	1	H	–	–	TCA
**tRNA^Ala^**	6307–6375	69	1	L	–	–	TGC
**tRNA^Asn^**	6384–6456	73	0	L	–	–	GTT
**tRNA^Cys^**	6457–6523	67	-1	L	–	–	GCA
**tRNA^Tyr^**	6523–6593	71	1	L	–	–	TGA
**COX1**	6595–8145	1551	-9	H	GTG	AGG	–
**tRNA^Ser(UCN)^**	8137–8209	73	3	L	–	–	GTC
**tRNA^Asp^**	8213–8281	69	8	H	–	–	GTC
**COX2**	8290–8973	684	1	H	ATG	TAA	–
**tRNA^Lys^**	8975–9043	69	1	H	–	–	TTT
**ATPase8**	9045–9212	168	-10	H	ATG	TAA	–
**ATPase6**	9203–9886	684	7	H	ATG	TAA	–
**COX3**	9894–10677	784	0	H	ATG	T--	–
**tRNA^Gly^**	10678–10746	69	0	H	–	–	TCC
**ND3**	10747–11097	351	1	H	ATG	TAA	
**tRNA^Arg^**	11099–11168	70	1	H	–	–	TCG
**ND4L**	11170–11466	297	-7	H	ATG	TAA	–
**ND4**	11460–12837	1378	0	H	ATG	TAT	–
**tRNA^His^**	12838–12907	70	0	H	–	–	GTG
**tRNA^Ser(AGY)^**	12908–12973	66	-1	H	–	–	GCT
**tRNA^Leu(CUN)^**	12973–13043	71	0	H	–	–	TAG
**ND5**	13044–14861	1818	8	H	ATG	AGA	–
**CYTB**	14870–16012	1143	5	H	ATG	TAA	–
**tRNA^Thr^**	16018–16086	69	18	H	–	–	TGT
**tRNA^Pro^**	16105–16174	70	6	L	–	–	TGG
**ND6**	16181–16699	519	-71	L	ATG	TAG	–
**tRNA^Glu^**	16701–16771	71	1	L	–	–	TTC

The length of the complete mitogenome of *E. personata* is 16,771 bp, and is similar to that of other Fringillidae species (Table 2). The base composition of the genome is C (32.1%), A (30.7%), T (23.0%) and G (14.2%); the proportion of A+T (53.7%) is higher than G+C (46.3%), suggesting a strong A+T bias. The mitogenomes of 17 Fringillidae species showed a positive AT-skew and a negative GC-skew.

**Table 2.** Base composition (in percentages) of the mitochondrial genomes of 17 species of Fringillidae.

Species	Total length (bp)	T (%)	C (%)	A (%)	G (%)	A + T content (%)	AT-skew	GC-skew	Accession number
***Eophona personata***	16771	23.0	32.1	30.7	14.2	53.7	0.142	-0.386	KX812499
***Eophona migratoria***	16798	22.9	32.3	30.7	14.0	53.7	0.145	-0.397	KX423959
***Oreomystis bairdi***	16833	23.7	31.6	30.3	14.4	53.9	0.123	-0.373	KM078807
***Paroreomyza montana***	16832	23.5	31.5	30.8	14.2	54.4	0.134	-0.379	KM078771
***Melamprosops phaeosoma***	16840	24.2	31.0	30.5	14.3	54.7	0.114	-0.370	NC_025617
***Acanthis flammea***	16820	24.0	31.4	30.5	14.2	54.5	0.120	-0.378	NC_027285
***Loxops coccineus***	15589	24.2	31.9	30.1	13.8	54.3	0.108	-0.395	KM078785
***Loxia curvirostra***	16805	23.8	31.4	30.6	14.3	54.3	0.125	-0.375	KM078800
***Carduelis spinus***	16828	24.0	31.3	30.9	13.8	54.9	0.127	-0.388	HQ915866
***Chloris sinica***	16813	24.7	30.5	30.7	14.1	55.4	0.108	-0.369	HQ915865
***Serinus canaria***	16805	23.9	31.1	31.1	13.8	55.0	0.130	-0.384	KM078794
***Haemorhous cassinii***	16812	24.3	30.5	30.9	14.2	55.2	0.120	-0.364	KM078786
***Coccothraustes coccothraustes***	16823	23.9	31.0	30.9	14.3	54.8	0.127	-0.369	KM078789
***Hemignathus parvu***s	16833	23.8	31.5	30.3	14.4	54.1	0.120	-0.371	KM078799
***Fringilla montifringilla***	16807	23.3	32.1	30.3	14.3	53.6	0.130	-0.382	JQ922259
***Hesperiphona vespertina***	16810	23.6	31.6	30.8	14.0	54.4	0.132	-0.387	KM078770
***Crithagra dorsostriata***	16804	24.2	30.8	31.1	13.8	55.4	0.125	-0.382	KM078798

Sequence analysis of the 13 PCGs in the mitogenome of *E. personata* revealed that the base composition of the *ND6* gene was not consistent with the other genes, and the percentage of T and G is much higher than in the other genes, with a positive GC skew (Table 3), whereas the base composition and skewness are highly similar for the other genes. The encoded genes share the common start codon ATN, with ATG most commonly observed. However, the start codon for COI is GTG. The non-coding regions include a control region (D-loop) and a few intergenic spacers. The control region is 1187 bp and is located between the tRNA^Glu^ and tRNA^Phe^.

**Table 3.** Base composition (in percentages) of the genes of *Eophona personata*.

**Gene**	**Proportion of nucleotides**				%**A+T**	**AT skew**	**GC skew**	%**A+C**	%**G+T**
	**T**	**C**	**A**	**G**					
**ND1**	25.3	33.3	27.3	14.1	52.6	0.039	-0.405	60.6	39.4
**ND2**	23.5	35.7	30.8	10.1	54.2	0.135	-0.559	66.4	33.6
**COX1**	23.3	32.7	27.5	16.5	50.8	0.081	-0.328	60.1	39.9
**COX2**	20.9	33.6	30.3	15.2	51.2	0.183	-0.377	63.9	36.1
**ATP8**	22.0	39.9	32.1	6.0	54.2	0.187	-0.740	72.0	28.0
**ATP6**	23.1	36.8	30.1	9.9	53.2	0.132	-0.575	67.0	33.0
**COX3**	24.0	33.3	27.6	15.2	51.5	0.069	-0.374	60.8	39.2
**ND3**	26.8	34.2	27.4	11.7	54.1	0.011	-0.491	61.5	38.5
**ND4L**	24.6	36.7	27.3	11.4	51.9	0.052	-0.524	64.0	36.0
**ND4**	23.1	35.6	30.5	10.9	53.6	0.138	-0.531	66.0	34.0
**ND5**	23.1	33.5	31.6	11.8	54.7	0.156	-0.480	65.1	34.9
**CYTB**	23.8	35.1	27.6	13.6	51.4	0.073	-0.442	62.6	37.4
**ND6**	38.9	9.6	10.4	41.0	49.3	-0.578	0.620	20.0	80.0
**12S rRNA**	20.0	27.7	31.4	20.9	51.4	0.222	-0.139	59.1	40.9
**16S rRNA**	21.4	24.2	34.7	19.7	56.1	0.236	-0.102	58.9	41.1
Total	23.6	31.9	29.3	15.2	52.9	0.108	-0.353	61.2	38.8

**Figure 2.**
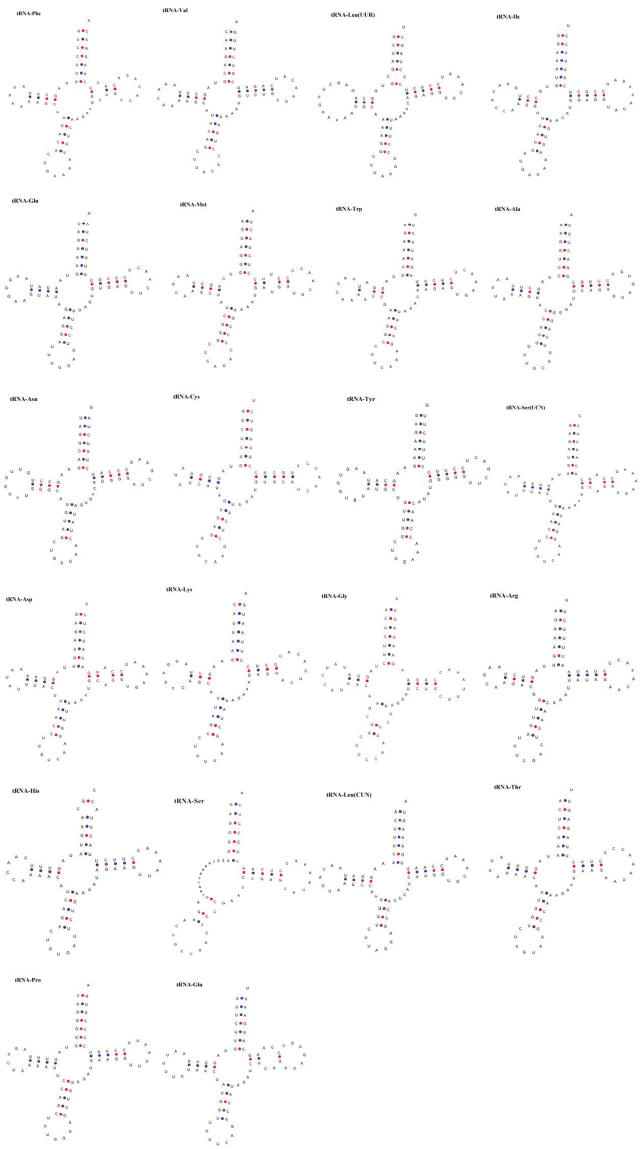
Predicted secondary structures for the 22 tRNAs in *Eophona personata*.

Based on the tRNA gene sequences identified, secondary structures of the tRNAs were determined (Fig. 2).

### Protein-coding genes and gene order

**Figure 3.**
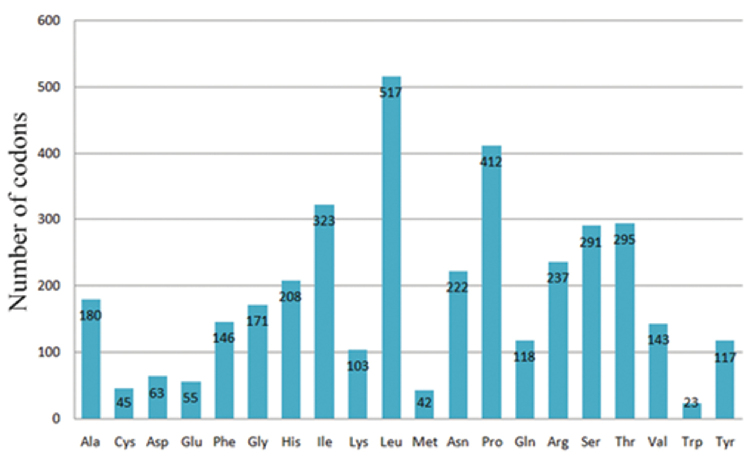
Codon distribution in the mitochondrial genome of *Eophona personata*.

The 13 PCGs in the mitogenome of *E. personata* spans a length of 11,399 bp, and encode for six NADH dehydrogenase subunits, three cytochrome c oxidase subunits, two ATPases and cytochrome b. The light-strand has nine genes, which includes eight tRNA genes and *ND6*, and the heavy-strand has 28 genes, which includes 14 tRNA genes, two rRNA genes and 12 protein-coding genes. Relative synonymous codon usage (RSCU) values for the 13 PCGs are shown in Table 4. There are 3798 codons in the 13 PCGs, and codons of leucine, proline, isoleucine, and threonine take a higher proportion (Fig. 3). The codons AAA-lysine, GAA-glutamic acid, AAC-asparagine, UUC-phenylalanine, and AAU-asparagine are AT-rich, and the codons CUC-leucine, CCU-proline, GCC-alanine, and AGC-serine are GC-rich.

**Table 4.** Relative synonymous codon usage (RSCU) values for the 13 protein-coding genes in *Eophona personata*.

Codon	Count	RSCU	Codon	Count	RSCU
UUU(F)	41	0.56	UCU(S)	40	0.82
UUC(F)	105	1.44	UCC(S)	55	1.13
UUA(L)	32	0.37	UCA(S)	52	1.07
UUG(L)	8	0.09	UCG(S)	8	0.16
CUU(L)	83	0.96	CCU(P)	174	1.69
CUC(L)	162	1.88	CCC(P)	114	1.11
CUA(L)	192	2.23	CCA(P)	105	1.02
CUG(L)	40	0.46	CCG(P)	19	0.18
AUU(I)	80	0.74	ACU(T)	99	1.34
AUC(I)	154	1.43	ACC(T)	92	1.25
AUA(I)	89	0.83	ACA(T)	94	1.27
AUG(M)	42	1.00	ACG(T)	10	0.14
GUU(V)	36	1.01	GCU(A)	39	0.87
GUC(V)	42	1.17	GCC(A)	88	1.96
GUA(V)	50	1.40	GCA(A)	46	1.02
GUG(V)	15	0.41	GCG(A)	7	0.16
UAU(Y)	41	0.70	UGU(C)	17	0.76
UAC(Y)	76	1.30	UGC(C)	28	1.24
UAA(*)	17	0.59	UGA(*)	67	2.31
UAG(*)	3	0.10	UGG(W)	23	1.00
CAU(H)	97	0.93	CGU(R)	27	0.68
CAC(H)	111	1.07	CGC(R)	54	1.37
CAA(Q)	102	1.73	CGA(R)	40	1.01
CAG(Q)	16	0.27	CGG(R)	28	0.71
AAU(N)	109	0.98	AGU(S)	39	0.80
AAC(N)	113	1.02	AGC(S)	97	2.00
AAA(K)	89	1.73	AGA(R)	35	0.89
AAG(K)	14	0.27	AGG(R)	53	1.34
GAU(D)	19	0.60	GGU(G)	34	0.80
GAC(D)	44	1.40	GGC(G)	53	1.24
GAA(E)	49	1.78	GGA(G)	61	1.43
GAG(E)	6	0.22	GGG(G)	23	0.54

**Figure 4.**
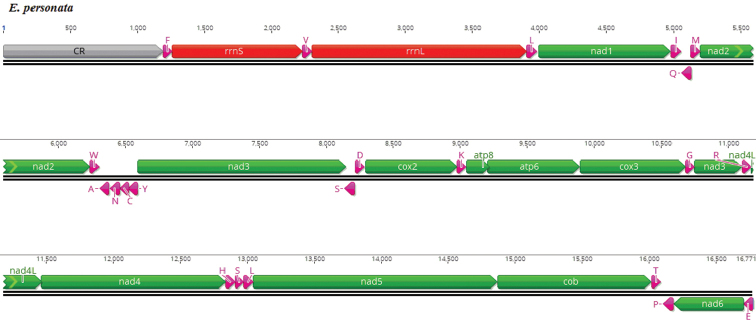
Mitochondrial gene order and arrangement in *Eophona personata*.

The gene order and arrangement of the region located between *CYTB* and tRNA^Phe^, is tRNA^Thr^, tRNA^Pro^, ND6, tRNA^Glu^, control region, and tRNA^Phe^, as shown in Fig. 4.

### Phylogenetic analysis

**Figure 5.**
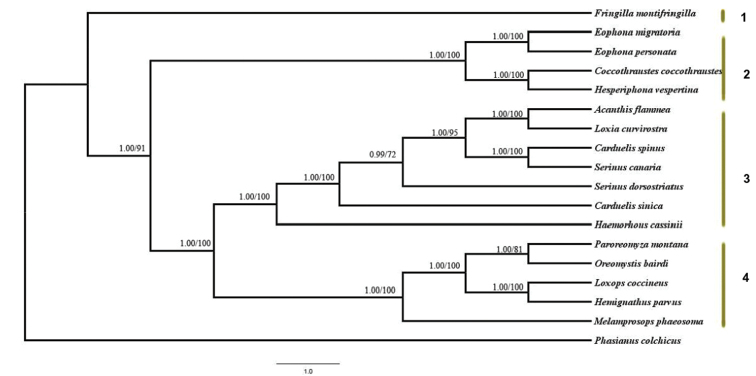
The phylogenetic tree generated for 17 species of Fringillidae. The values indicated at the nodes are Bayesian posterior probabilities (left) and ML bootstrap proportions (right).

Phylogenetic relationships of the 17 Fringillidae species, inferred from Bayesian and Maximum Likelihood analyses, recovered almost identical well-resolved topologies (Fig. 5). Four major clades can be distinguished: clade 1 consisted of the genus *Fringilla*; clade 2 comprised three genera of grosbeaks (*Coccothraustes*, *Eophona*, *Hesperiphona*); clade 3 comprised of five genera (*Acanthis*, *Loxia*, *Carduelis*, *Serinus*, and *Haemorhous*); and clade 4 consisted of three genera of honeycreepers (*Hemignathus*, *Loxops*, and *Paroreomyza*). Clades 3 and 4 were inferred as sister-groups, which together were sister to clade 2. Clade 1 was sister to all other cardeline finches.

The analysis recovered *C. coccothraustes* and *H. vespertina* as sister groups to *Eophona*, with strong support in both Bayesian and ML analysis (Fig. 5).

No evidence for chimerism was found in comparisons of the ND2, COX1, and CYTB fragments with reference sequences on GenBank. Thus, in all cases the mitogenome of *E. personata* clustered with, and was very similar to, reference sequences of this species (data not shown).

## Discussion

In this study, we obtained the complete mitogenome sequence of *E. personata*, and performed molecular phylogenetic analysis of 17 Fringillidae species based on the sequences of 12 mitochondrial PCGs. Our results revealed that the complete mitogenome of *E. personata* is 16,771 bp, and contains 13 protein-coding genes, 22 transfer RNA (tRNA) genes, two ribosomal RNA (rRNA) genes, and one control region. Analysis of the base composition revealed an A+T bias, a positive AT skew and a negative GC skew. Phylogenetic analysis demonstrated that *Coccothraustes* is the closest genus to *Eophona* and that *E. personata* is the sister taxon of *E. migratoria*.

Our analysis showed that the mitogenome of *E. personata* is similar to that of other Fringillidae species. The genome structure was that of a typical vertebrate mitochondrial genome (Boore 1999). The A+T bias observed in the mitochondrial genome of *E. personata* was similar to that of other vertebrates (Yang et al. 2016), and the positive AT-skew value and a negative GC-skew value observed, is also consistent with the vertebrate mitogenome (Quinn and Wilson 1993). The gene order and arrangement in *E. personata* was similar to the typical arrangement seen in birds (Mindell et al. 1999; Haddrath and Baker 2001; Liu et al. 2013), including Passeriformes (Caparroz et al. 2018). It is generally believed that the rearrangement of the mitochondrial genome represents a rare evolutionary event that can be used to construct the phylogenetic relationships of distantly related groups (Bensch 2000). In birds, two major types of gene order are found which differ by the number of control region copies (Singh et al. 2008). One of these is believed to be the ancestral gene order and the other is the remnant control region 2 gene order (Singh et al. 2008).

The phylogenetic relationships observed in this study are in accordance with previous research (Arnaiz-Villena et al. 2001; Zuccon et al. 2012). In the current study, the three grosbeak genera (*Coccothraustes*, *Eophona*, *Hesperiphona*; clade 2) clustered together and formed a well-defined clade. The grosbeak consists of a group of fairly large and stocky finches, feeding primarily on hard seeds. Our study corroborates a previous study based on the nuclear and mitochondrial sequences (Zuccon et al. 2012). A close phylogenetic relationship between the four genera (*Coccothraustes*, *Mycerobas*, *Hesperiphona*, *Eophona*) has been recognised previously (Vaurie 1959; Clement et al. 1993; Dickinson and Christidis 2015), and their close evolutionary relationships are widely accepted.

Our analysis showed that the genus *Fringilla* diverged very early within the family Fringillidae and was followed a deep divergence between the grosbeak clade and a clade formed by the other members Carduelinae. Our study as well as previous studies suggests that the analysis of phylogenetic relationships of passerines are more accurately resolved and better supported with complete mitogenomes than with short sequences of single genes (Van der Meij et al. 2005; Nguembock et al. 2009).

## Conclusions

In this study, the complete mitogenome of *E. personata* was sequenced and analysed for the first time, and the phylogenetic analysis confirmed the taxonomic classification of *E. personata*. The results showed that the genera *Coccothraustes* and *Hesperiphona* have a close relationship with the genus *Eophona*, and this is consistent with the morphologicl similarity observed between them. Our analysis shows the phylogenetic relationship of *E. personata* as a sister group to *E. migratoria*, and the mitogenome was observed to be very similar between them.
